# PutA Is Required for Virulence and Regulated by PruR in *Pseudomonas aeruginosa*

**DOI:** 10.3389/fmicb.2018.00548

**Published:** 2018-03-26

**Authors:** Ruiping Zheng, Xuemei Feng, Xueying Wei, Xiaolei Pan, Chang Liu, Ruopu Song, Yongxin Jin, Fang Bai, Shouguang Jin, Weihui Wu, Zhihui Cheng

**Affiliations:** ^1^Department of Microbiology, College of Life Sciences, Nankai University, Tianjin, China; ^2^Department of Molecular Genetics and Microbiology, College of Medicine, University of Florida, Gainesville, FL, United States

**Keywords:** *Pseudomonas aeruginosa*, PutA, PruR, bacterial virulence, gene regulation

## Abstract

*Pseudomonas aeruginosa*, a Gram-negative opportunistic pathogenic bacterium, causes acute and chronic infections. Upon entering the host, *P. aeruginosa* alters global gene expression to adapt to host environment and avoid clearance by the host immune system. Proline utilization A (PutA) is a bifunctional enzyme, which converts proline to glutamate. Here we report that PutA was required for the virulence of *P. aeruginosa* in a murine acute pneumonia model. A *putA* mutant was more susceptible to oxidative stress compared to the wild type strain. An AraC/XylS family protein, PruR, directly bound to the upstream of −35 box in the *putA* promoter and activated *putA* expression. High concentration of proline in bacteria up-regulated *pruR* expression, which led to the activation of *putA* expression. As a feedback regulation, glutamate produced by PutA released PruR from the *putA* promoter and turned off the *putA* expression. PruR affected bacterial virulence through the regulation of the *putA* expression. Altogether, these data are the first to reveal that PutA plays an important role in the pathogenesis of *P. aeruginosa*, as well as to describe the genetic regulation of PutA in *P. aeruginosa*.

## Introduction

*Pseudomonas aeruginosa* is a wide-spread Gram-negative opportunistic human pathogen, which causes acute and chronic infections, such as pneumonia, severe burn infections, sepsis, and urinary tract infections (Williams et al., 2010; Gellatly and Hancock, 2013). During infection, *P. aeruginosa* regulates the expression of a variety of virulence factors to counteract host immune defense and increase tolerance against antibiotics, such as type III secretion system (T3SS), iron acquisition, quorum sensing system, biofilm formation, and multidrug efflux pumps (Lister et al., 2009; Turner et al., 2014; Goo et al., 2015; Rybtke et al., 2015; Anantharajah et al., 2016; Huber et al., 2016; Reinhart and Oglesby-Sherrouse, 2016).

In a mouse acute pneumonia model, neutrophils are rapidly recruited to the infection site in response to the invading bacteria (Shaver and Hauser, 2004). Neutrophils are the major phagocytes that generate reactive oxygen species (ROS) to kill bacteria (Ziltener et al., 2016). In *P. aeruginosa*, the H_2_O_2_-responsive regulator OxyR senses oxidative stress and activates the expression of defensive genes, such as *katA* (catalase A), *katB* (catalase B), *ahpB* (alkyl hydroperoxide reductases B), and *ahpCF* (alkyl hydroperoxide reductases CF), to breakdown the ROS (Ochsner et al., 2000; Heo et al., 2010).

Proline is an important carbon and nitrogen source for bacterial growth (Wood, 1981; Kohl et al., 1988; Nagata et al., 2003). It also provides protection against osmotic, heat and oxidative stresses in prokaryotes as well as in eukaryotes (Csonka, 1981b; Chattopadhyay et al., 2004; Chen and Dickman, 2005; Natarajan et al., 2012). In *Escherichia coli* and *Colletotrichum trifolii*, proline utilization increases oxidative stress resistance by up-regulation of catalase expression (Chen and Dickman, 2005; Zhang et al., 2015).

Proline utilization is a successive two-step process converting proline to glutamate, which is coordinated by proline dehydrogenase (PRODH) and 1-pyrroline-5-carboxylate dehydrogenase (P5CDH) (Tanner, 2008). In eukaryotes, such as humans, plants and animals, the PRODH and P5CDH are two separate enzymes in the mitochondrion, however in certain Gram-negative bacteria, such as *Helicobacter pylori*, PRODH and P5CDH are combined into a bifunctional enzyme, named proline utilization A (PutA) (Singh and Tanner, 2012). It has been shown that PutA plays a critical role in bacterial pathogenesis. PutA mutant strains showed less efficiency in the colonization of mice than the wild-type strains in *H. pylori, Helicobacter hepaticus*, and *Brucella abortus* (Krishnan et al., 2008; Nakajima et al., 2008; Caudill et al., 2017). However, contribution of the PutA on the pathogenecity of *P. aeruginosa* is not known.

**Figure 1:**
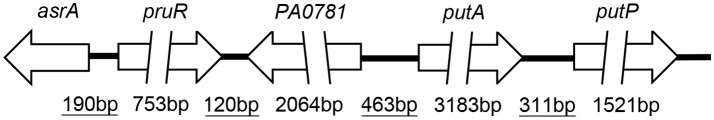
Schematic diagram of *putA* gene in *P. aeruginosa*. Genes are represented by open arrows. The gene designations are above the arrows. The length of each gene is indicated below the arrow. The length of each intergenic region is indicated below the bar and underlined. Data were from *P. aeruginosa* reference strain PAO1 (Winsor et al., 2016). The length of each gene and intergenic region is conserved between the PAO1 strain and *P. aeruginosa* PAK strain. The homology of the fragment (from the intergenic region of *asrA* and *pruR* gene to the *putP* gene, 8,605 bp) between the PAO1 strain and the PAK strain is 99.78%.

In *E. coli, Salmonella typhimurium*, or *Pseudomonas putida*, PutA is about 1,300 amino acid residue long, containing three domains, PRODH domain, P5CDH domain, and an additional N-terminal ribbon-helix-helix (RHH) DNA binding domain, and acts as a transcriptional repressor (Hahn et al., 1988; Vílchez et al., 2000; Zhou et al., 2008a). When intracellular proline level is low, PutA binds to operator sites located between two divergently transcribed genes, *putA* and *putP* (encoding a proline permease), and represses their expression (Zhou et al., 2008a,b). When proline level is high, PutA dissociates from the binding sites and moves to the inner membrane, which activates the Put system expression (Zhou et al., 2008a,b). In *P. aeruginosa*, PutA is 1,060 amino acid residue long and contains only two domains, PRODH domain and P5CDH domain. And *putA* and *putP* form an operon (Nakada et al., 2002; Figure 1). It is unlikely that PutA functions as a transcriptional repressor to regulate the expression of Put system in *P. aeruginosa*. In *Agrobacterium tumefaciens* and *Rhodobacter capsulatus*, the expression of *putA* is trans-activated by a Lrp-family protein, called PutR (Keuntje et al., 1995; Cho and Winans, 1996). In *Ehrlichia chaffeensis*, a two-component system NtrY/NtrX up-regulates *putA* expression upon bacterial entry into host cells (Cheng et al., 2014). However, these genes are not present in the *P. aeruginosa* genome. In *P. aeruginosa, pruR* encoding an AraC/XylS family protein is located close to the *putAP* operon (Figure 1). The expression of *putA* gene is down-regulated in a Δ*pruR* mutant strain, suggesting *putA* expression is regulated by the PruR (Nakada et al., 2002). Until now, no definitive analysis of the regulatory mechanisms by which *P. aeruginosa* can modulate *putA* expression has been reported.

In this article, to gain insights into the role of PutA in *P. aeruginosa* virulence, we constructed a Δ*putA* mutant and determined its mortality in an acute pneumonia model. We also determined the regulatory mechanisms of *putA* expression by *lacZ* reporter assays, electrophoretic mobility shift assays and DNase I footprint assays. Overall, these data provide important information about the function and regulation of PutA in *P. aeruginosa*.

## Materials and methods

### Ethics statement

All animal studies complied with National and Nankai University guidelines regarding the use of animals in research. All animal experiment protocols have been approved by the institutional animal care and use committee of the College of Life Sciences of Nankai University (permit number NK-04-2012).

### Bacterial strains and plasmids

The bacterial strains used in this study are listed in Table S1. *E. coli* strains DH5α and S17-1 used for general cloning and conjugal transferring, respectively, were cultured in Luria–Bertani (LB) broth (10 g/l tryptone [Oxoid Ltd., Basingstoke, UK], 5 g/l NaCl [Sangon Biotech, Shanghai, China], 5 g/l yeast extract [Oxoid Ltd.], pH 7.0–7.5) or LB agar (LB broth containing 15 g/l agar [BBI life sciences, Shanghai, China]) under aerobic condition at 37°C. *P. aeruginosa* strains were cultured in LB broth or minimal medium P (MMP) supplemented with different concentration of proline (Oxoid Ltd.) or glutamate (Oxoid Ltd.). MMP was composed of basal salt solution (BBS) supplemented with 1.0% (w/v) glucose (Sangon Biotech) and 0.1% (w/v) (NH_4_)_2_SO_4_ (BBI life sciences). BBS was composed of 2% buffer solution (7.3% [w/v] Na_2_HPO_4_ [Sangon Biotech], 3.2% [w/v] KH_2_PO_4_ [Sangon Biotech], pH 7.2), 40 g/l MgSO_4_ (BBI life sciences), and 4 g/l FeSO_4_ (BBI life sciences) (Haas et al., 1977). When needed, the medium was supplemented with tetracycline (50 μg/ml) (BBI life sciences), gentamicin (100 μg/ml) (BBI life sciences), carbenicillin (150 μg/ml) (BBI life sciences), kanamycin (30 μg/ml) (BBI life sciences), or ampicillin (100 μg/ml) (BBI life sciences).

Plasmids used in this study are listed in Table S2. For DNA manipulation, standard protocols or manufacture instructions of commercial products were followed. Chromosomal gene mutation strains, Δ*putA* and Δ*pruR*, were generated as described previously (Hoang et al., 1998). Complemented strains, Δ*putA/ putA* and Δ*pruR/pruR* were obtained by integrating each gene including its promoter region into chromosome using pUC18T-mini-Tn7T-Gm (Choi and Schweizer, 2006). Insertion of an empty transposon (with the inverted repeats only) was not included, since previous researches have demenstrated that insertion in chromosome using this method did not change the characters of bacteria (Choi and Schweizer, 2006; Heacock-Kang et al., 2017; Munguia et al., 2017; Pletzer et al., 2017). Primers used for knock-out and complementation are listed in Table S3.

### Murine acute pneumonia model

Bacteria were grown in LB broth at 37°C for overnight and then sub-cultured into fresh LB broth at 37°C with aeration to OD_600_ of 1.0. Bacterial cells were harvested by centrifugation and adjusted to 2 × 10^9^ CFU/ml in sterile 1 × PBS (274 mM NaCl, 5.4 mM KCl [BBI life sciences], 20 mM Na_2_HPO_4_, 4 mM KH_2_PO_4_, pH 7.4). Female BALB/c mice (6- to 8-week old) (Academy of Military Medical Sciences, Beijing, China) were anesthetized with an intraperitoneal injection of 7.5% chloral hydrate (70 μl per mouse). Anesthetized mice were intranasally inoculated with 10 μl of bacterial suspension in each nostril, giving a total infection volume of 20 μl. Thirteen mice were used for each strain. The mice were monitored at least twice a day for 5 days (Weng et al., 2016).

### H_2_O_2_ susceptibility assay

H_2_O_2_ susceptibility assay was performed as described with minor modification (Weng et al., 2016). Over-night cultures of *P. aeruginosa* strains were diluted in the indicated medium to OD_600_ of 0.05 and cultured at 37°C. When OD_600_ reached 1.0, bacterial cells from 3 ml culture were collected and washed twice with sterile 1 × PBS. Then the bacterial cells were resuspended in 1 × PBS with 0.3% H_2_O_2_ and incubated at 37°C for 15 min. The live bacterial numbers were determined by serial dilution and plating.

### Expression and purification of recombinant protein

The DNA fragment encoding full-length PruR was amplified by PCR using *P. aeruginosa* PAK chromosomal DNA as a template and specific primers (Table S3). The amplified fragment was digested with restriction enzymes and ligated into the same restriction enzyme-digested pET-28a(+). *E. coli* DH-5α cells were transformed with the ligation product. Plasmids were extracted using an Axyprep Plasmid Miniprep Kit (Axygen Biosciences, CA, USA) and the cloned fragment was confirmed by DNA sequencing. *E. coli* BL21(DE3) cells were then transformed with the resulting plasmid and induced to express the recombinant protein with isopropyl-thio-ß-D-galactoside (IPTG). The recombinant PruR (rPruR) were Ni-affinity purified from *E. coli* soluble fraction (Cheng et al., 2006). The purified protein was dialyzed against stocking buffer (50 mM Tris [Genview, Beijing, China], pH 7.9, 50 mM NaCl, 0.5 mM EDTA [Solarbio, Beijing, China], 10% [v/v] glycerol [Solarbio]).

### Construction of *lacZ* fusions

For reporter assay in *E. coli, lacZ* fusions were constructed as described (Cheng et al., 2008). Briefly, DNA fragments were amplified by PCR using specific primers (Table S3) and inserted upstream of the promoter-less *lacZ* gene in pACYC184 (New England Biolabs, MA, USA). BL21(DE3) strain containing pET-28a(+) encoding rRruR, or pET-28a(+) alone (negative control) was transformed with the *lacZ* fusion constructs. After inducing the recombinant protein with 0.1 mM IPTG at 37°C for 2 h, β-galactosidase activity was measured as described previously (Wang et al., 2007). Recombinant protein expression was confirmed by Western blot analysis using the anti-His-tag antibody (Sigma, MO, USA).

For reporter assay in *P. aeruginosa*, DNA fragment was amplified by PCR with specific primers (Table S3) and inserted upstream of the promoter-less *lacZ* gene in pDN19*lacZ*Ω. *P. aeruginosa* strains were transformed with the *lacZ* fusion construct or pDN19*lacZ*Ω vector (negative control). β-galactosidase activity was measured as described (Weng et al., 2016).

### Electrophoretic mobility shift assay

Electrophoretic mobility shift assay (EMSA) was performed as described with minor modification (Cheng et al., 2008). Briefly, DNA fragments corresponding to the sequences upstream of *putA* were amplified by PCR using specific primers (Table S3). DNA fragments (50 ng) were incubated with 2 μg purified rPruR in a 20-μl reaction (50 mM Tris, pH 7.9, 50 mM NaCl, 0.5 mM EDTA, 10% glycerol, 1% [v/v] NP-40 [Solarbio]) at 25°C for 10 min. Samples were loaded onto an 8% native polyacrylamide gel in 1 × Tris-borate-EDTA (TBE) buffer (0.044 M Tris, 0.044 M boric acid, 0.001 M EDTA, pH 8.0) that had been prerun for 1 h, electrophoresed on ice at 100 V for 1.5 h, followed by DNA staining in 1 × TBE containing 0.5 μg/ml ethidium bromide. Bands were visualized with a molecular imager ChemiDoc TM XRS+ (Bio-Rad, CA, USA).

### DNase I footprint analysis

DNase I footprint analysis was performed as described with minor modification (Zianni et al., 2006). A 184-bp DNA fragment upstream of the *putA* gene was amplified by PCR with primers shown in Table S3, except that the forward primer was labeled with 6-carboxyfluorescein (FAM). The FAM-labeled probe (300 ng) was incubated with 1 or 2 μg rPruR or 2 μg BSA (negative control) under the conditions described previously. Based on the results of DNase I (Takara, Dalian, China) optimization experiments, 0.09 U of DNase I was added to each reaction mixture and incubated at 25°C for 5 min. The reaction was terminated by heating the mixture at 80°C for 10 min. The digested DNA fragments were extracted with phenol:chloroform:isoamyl alcohol (25:24:1) and precipitated with ethanol. The pellets were dissolved in 20 μl water. The sequences were then analyzed with Peak Scanner software v1.0 (Applied Biosystems, CA, USA) to convert the DNase I digestion maps into sequencing data to identify the exact sequences that were protected.

### Proline concentration assay

Proline concentration in bacterial cells was determined as described (Shabnam et al., 2016). Harvested bacterial cells were resuspended in 150 μl water and boiled for 10 min. After centrifugation at 14,000 rpm for 5 min at 4°C, 100 μl of the supernatant was incubated with 100 μl of acid-ninhydrin (0.25 g ninhydrin dissolved in 6 ml glacial acetic acid and 4 ml 6 M phosphoric acid) and 100 μl of glacial acetic acid at 100°C for 1 h. The reaction was stopped by incubation on ice, and the mixture was extracted with 400 μl toluene. The toluene phase was separated, and the OD_520_ was measured to determine the concentration of proline in the extract.

### Quantitative RT-PCR

Total RNA was isolated from bacteria using an RNeasy Minikit (Tiangen Biotech, Beijing, China). The cDNA was synthesized from total RNA using random primers and PrimeScript Reverse Transcriptase (TaKaRa). For quantitative PCR, cDNA was mixed with 4 pmol of forward and reverse primers (Table S3) and SYBR Premix Ex TaqTM II (TaKaRa) in a total reaction volume of 20 μl. The results were determined using a CFX Connect Real-Time system (Bio-Rad).

### Statistical analysis

Statistical analyses were performed with the GraphPad Prism software. Survival data were analyzed with the log-rank (Mantel-Cox) test. Other results were analyzed by the Student's *t*-test (two-tailed).

## Results

### PutA is essential for *P. aeruginosa* infection in a mouse acute pneumonia model

**Figure 2:**
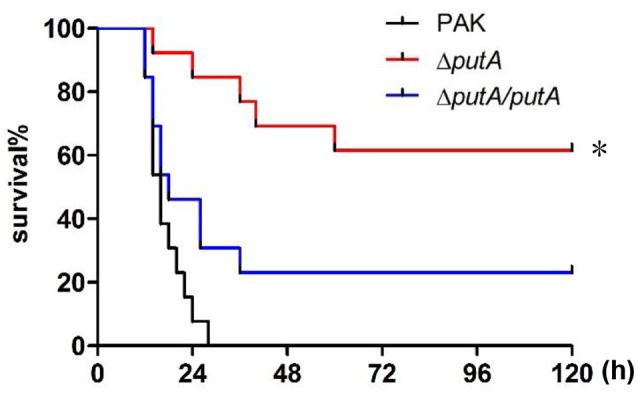
Role of PutA in *P. aeruginosa* infection in a mouse acute pneumonia model. Mice were inoculated intranasally with 4 × 10^7^ CFU bacteria of indicated strains. The mice were monitored for 5 days after the infection. The data were from 13 mice per strain. ^*^p < 0.01, compared to the wild type PAK strain or the Δ*putA*/*putA* complemented strain by log-rank (Mantel-Cox) test.

Given that little information is currently available about the role of the proline utilization system in *P. aeruginosa* infection, we sought to define the function of PutA by assessing the requirement of PutA for *P. aeruginosa* virulence. We infected mice with a wild type PAK strain (Weng et al., 2016), a Δ*putA* mutant strain or a Δ*putA*/*putA* complemented strain in an acute pneumonia model. Compared to the wild type PAK strain, the Δ*putA* mutant strain caused significantly lower mortality (*P* < 0.01; Figure 2). Complementation with a *putA* gene driven by its native promoter restored the bacterial virulence (Figure 2). The growth rates of these three strains showed no differences when cultured on LB broth or MMP medium supplemented with glucose and (NH_4_)_2_SO_4_ (Figure S1). These results indicated that PutA is required for the virulence of *P. aeruginosa* in the murine acute pneumonia model.

### *P. aeruginosa* Δ*putA* mutant strain is more sensitive to oxidative stress compared to the wild type PAK strain *in vitro*

**Figure 3:**
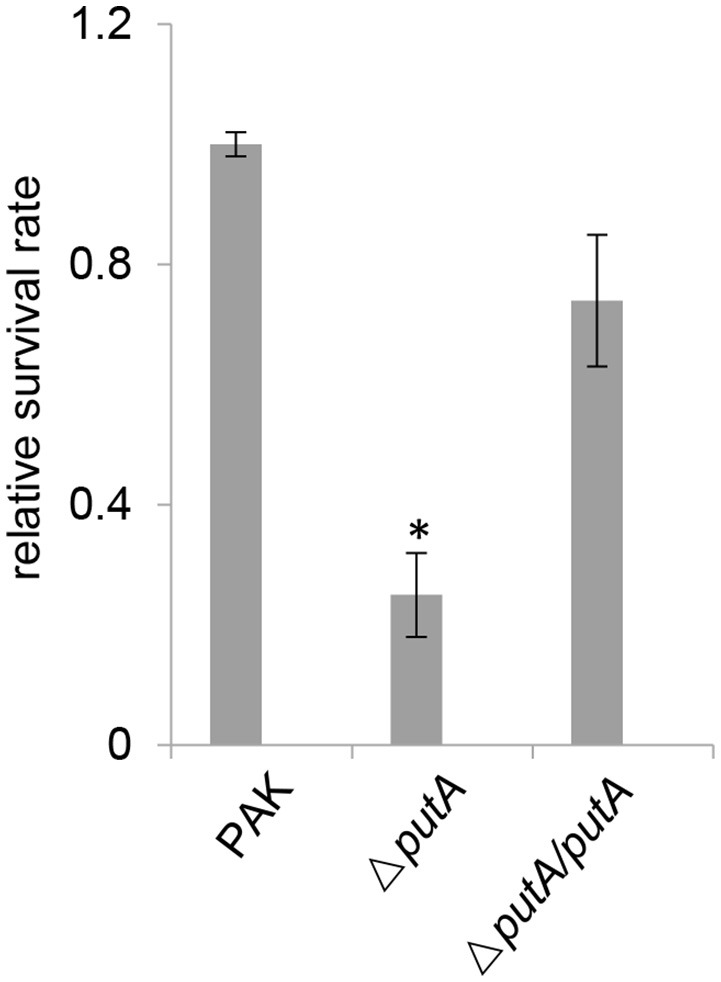
Roles of PutA in bacterial resistance to oxidative stress. Indicated strains were treated with 0.3% H_2_O_2_ at 37°C for 15 min and the numbers of live bacteria were determined by serial dilution and plating. The values reflect bacterial numbers of each strain relative to that of wild type PAK strain. Data indicate the means ± standard deviations from three independent experiments performed in triplicate. ^*^P < 0.01 compared to the wild type PAK strain or the Δ*putA*/*putA* complemented strain by student's *t*-test.

It has been shown previously that bacterial PutA are linked to the resistance against oxidative stress (Zhang et al., 2015). ROS produced by neutrophils is one of the major mechanisms of host defense against bacteria (Shaver and Hauser, 2004; Ziltener et al., 2016). To test the hypothesis that PutA in *P. aeruginosa* is required for the ability of bacteria to cope with oxidative stress, we treated the wild type PAK strain, the Δ*putA* mutant strain or the Δ*putA*/*putA* complemented strain with 0.3% H_2_O_2_ at 37°C for 15 min. Survival rate of the Δ*putA* mutant strain was significantly lower than that of wild type PAK strain (Figure 3). Complementation of *putA* gene rescued the mutant strain from the increased sensitivity to H_2_O_2_-mediated killing (Figure 3). These experiments indicated that proline utilization is involved in bacterial defense against oxidative stress, which aids bacteria to establish infection in hosts.

### PruR activates *putA* expression

**Figure 4:**
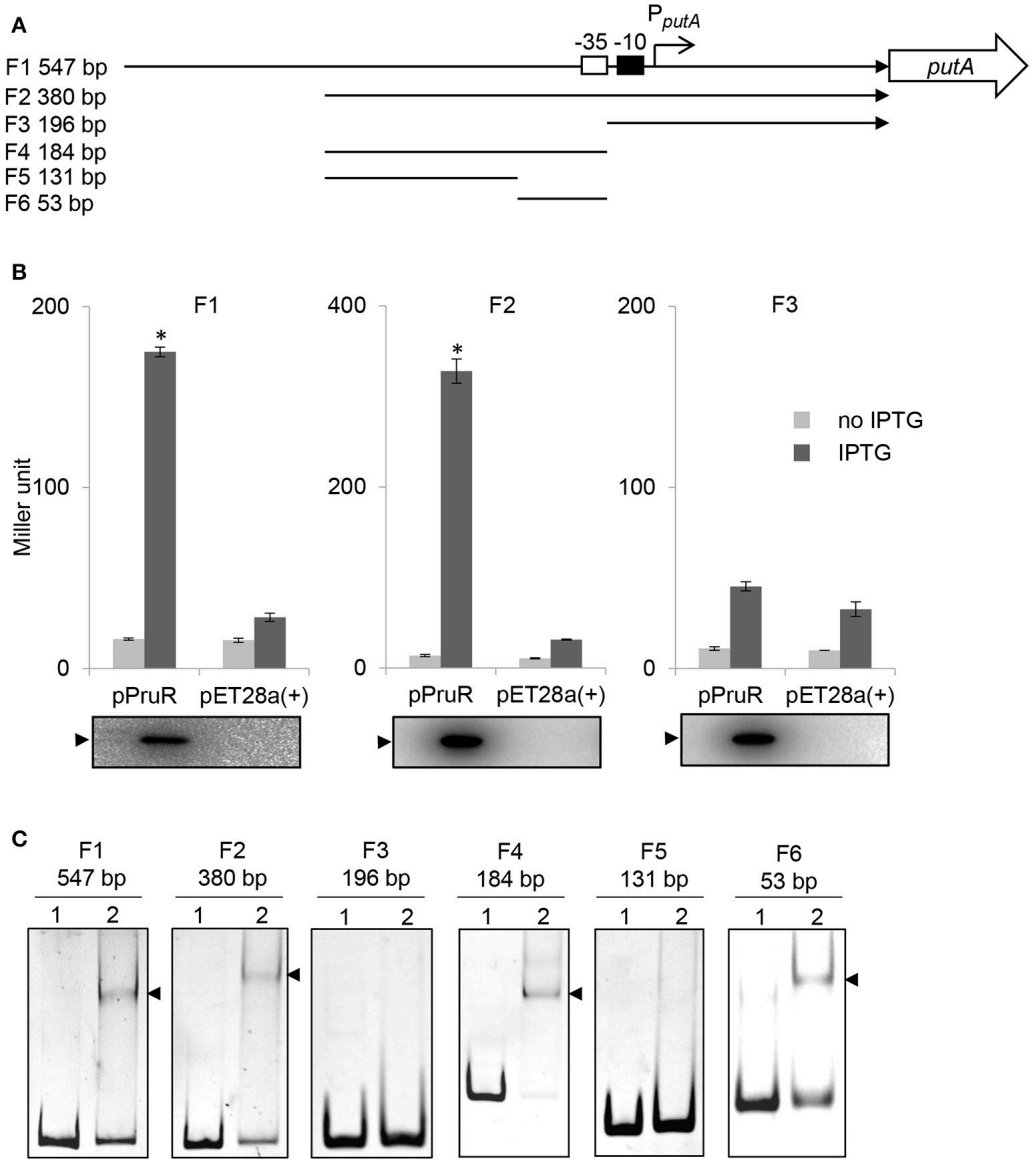
PruR activates the *putA* expression and directly binds to the *putA* promoter. **(A)** Schematic diagram of the fragments used for reporter assay and EMSA. The *putA* gene is represented by an open arrow. The transcriptional start site of *putA* gene is indicated by an arrow. −35 box (an open box) and −10 box (a solid box) are shown. Three fragments, F1-3, corresponding to various regions upstream of *putA* gene were inserted upstream of the promoter-less *lacZ* gene in pACYC184 for reporter gene assay. The direction of each fragment is indicated by an arrow. Six fragments, F1-6, corresponding to various regions upstream of *putA* gene were used for EMSA. Designation and length of each fragment is shown on the left. **(B)** PruR activates the *putA* expression. β-galactosidase assays were used to measure the transcriptional activities of *lacZ* reporter fusions. Data indicate the means ± standard deviations from three independent experiments performed in triplicate. ^*^P < 0.01 compared to no IPTG induction or the empty pET-28a(+) vector by student's *t*-test. Western blot analyses of samples from the β-galactosidase assays were performed using an anti-His-tag antibody to verify the expression of rPruR. The blots below the graphs are representative blots for three independent experiments. The position of rPruR is indicated by an arrowhead. **(C)** PruR binds to the *putA* promoter. Lane 1, DNA probe (50 ng); lane 2, DNA probe incubated with rPruR (2 μg). Shifted bands are indicated by arrowheads. The length (bp) of each fragment is shown above each panel. The gel was stained with ethidium bromide.

Since PutA is essential for *P. aeruginosa* virulence, we then investigated the regulation of *putA* expression. The only available information is that in a Δ*pruR* mutant *P. aeruginosa* strain, *putA* expression was down-regulated, suggesting *putA* expression is under the control of PruR (Nakada et al., 2002). We examined whether PruR transcriptionally activates the *putA* expression. Fragments (F1, F2, F3) derived from *putA* promoter region with various lengths were inserted upstream of a promoter-less *lacZ* gene in a pACYC184 plasmid (Figure 4A). The fusion plasmids were transformed into *E. coli* BL21(DE3) strain harboring a plasmid (pPruR) expressing recombinant *P. aeruginosa* PruR (rPruR) or an empty pET-28a(+) vector. Induction of rPruR by IPTG resulted in a significant increase in β-galactosidase activity in the strain harboring F1-*lacZ* fusion or F2-*lacZ* fusion, but not in the strain harboring F3-*lacZ* fusion (Figure 4B), indicating that PruR binds to F1 and F2 fragments of *putA* promoter and trans-activates *putA* expression. The expression of rPruR was confirmed by Western blot analysis (Figure 4B). Also we examined whether PruR activates the expression of *PA0781* gene, which is located upstream of the *putA* in diverging direction. Our β-galactosidase assay results showed PruR could not activate the expression of *PA0781* gene (Figure S2).

### PruR binds directly to the *putA* promoter

**Figure 5:**
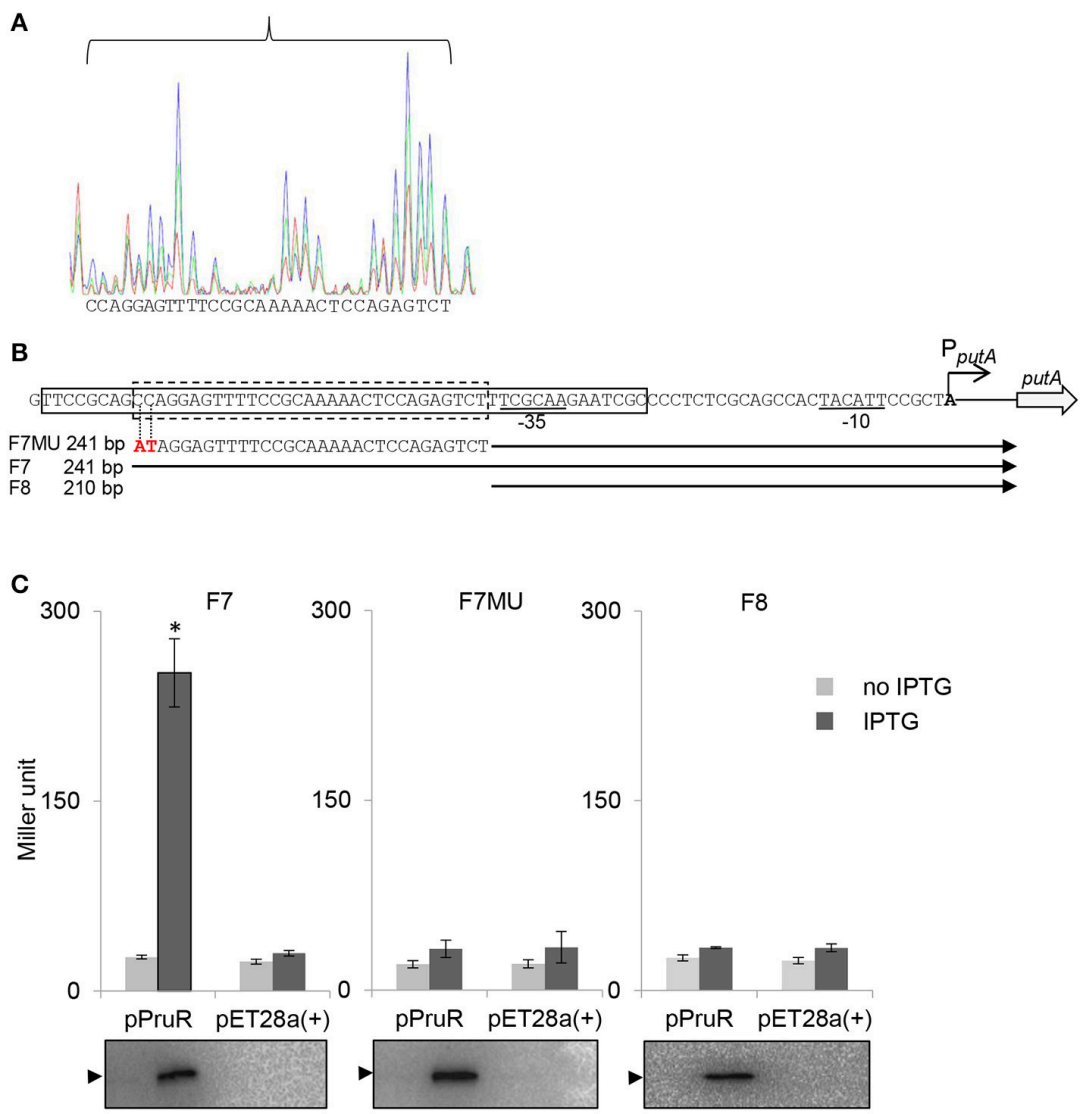
The PruR binding site is upstream of −35 box of the *putA* promoter. **(A)** Identification of the sequence of the PruR-protected region in the *putA* promoter by DNase I protection footprinting. Electropherograms are superimposed to show the region protected by different concentrations of rPruR (green, 1 μg; red, 2 μg;) or BSA (blue, 2 μg) within the *putA* promoter after digestion with DNase I. The DNA sequence protected by PruR is shown below the electropherograms. **(B)** The PruR binding site in the *putA* promoter. The sequence of F6 fragment is boxed with solid lines. The sequence protected by PruR is boxed with dash line. −35 box and −10 box are underlined. The transcriptional start site is bold and labeled with an arrow. Fragments F7, F7MU (the mutated nucleotides are in red letters and indicated by dotted lines), and F8 were inserted upstream of the promoter-less *lacZ* gene in pACYC184 for reporter gene assay. Designation and length of each fragment is shown on the left. **(C)** PruR binds to the protected region and activates the *putA* expression. β-galactosidase assays were used to measure the transcriptional activities of *lacZ* reporter fusions. Data indicate the means ± standard deviations from three independent experiments performed in triplicate. ^*^P < 0.01 compared to no IPTG induction or the empty pET-28a(+) vector by student's *t*-test. Western blot analyses of samples from the β-galactosidase assays were performed using an anti-His-tag antibody to verify the expression of rPruR. The blots below the graphs are representative blots for three independent experiments. The position of rPruR is indicated by an arrowhead.

To examine whether PruR directly binds to the *putA* promoter and determine the binding site, we preformed EMSA using fragments derived from different positions in the *putA* promoter region (Figure 4A). Upon incubation with rPruR, shifted bands were detected for fragment F1, F2, F4, and F6, but not for F3 and F5, indicating that PruR bound to the *putA* promoter region and the binding site was located in F6, whose location is from −79 to −27 counting from the transcriptional start site (Figures 4C, 5B).

Next we performed DNase I protection assays to reveal the DNA sequence to which PruR binds. The protected region of the sense strand was determined by comparing the sequence of a DNA sample protected by 1 or 2 μg rPruR to the sequence of an unprotected DNA sample incubated with 2 μg BSA (Figure 5A). The region protected by rPruR was located from −71 to −41 counting from the *putA* transcriptional start site, which was in F6 fragment and right upstream of −35 box (Figure 5B). To confirm the PruR binding site, fragments F7 (containing the binding site) and F8 (without the binding site) were inserted upstream of a promoter-less *lacZ* gene in a pACYC184 plasmid (Figure 5B). Also we mutated CC (−71, −70 from the transcriptional start site) into AT in F7-*lacZ* fusion (F7MU, Figure 5B). Induction of rPruR by IPTG resulted in a significant increase in β-galactosidase activity in the strain harboring F7-*lacZ* fusion, but not in the strain harboring F7MU-*lacZ* fusion or F8-*lacZ* fusion (Figure 5C).

### Proline and glutamate are signals controlling *putA* expression

**Figure 6:**
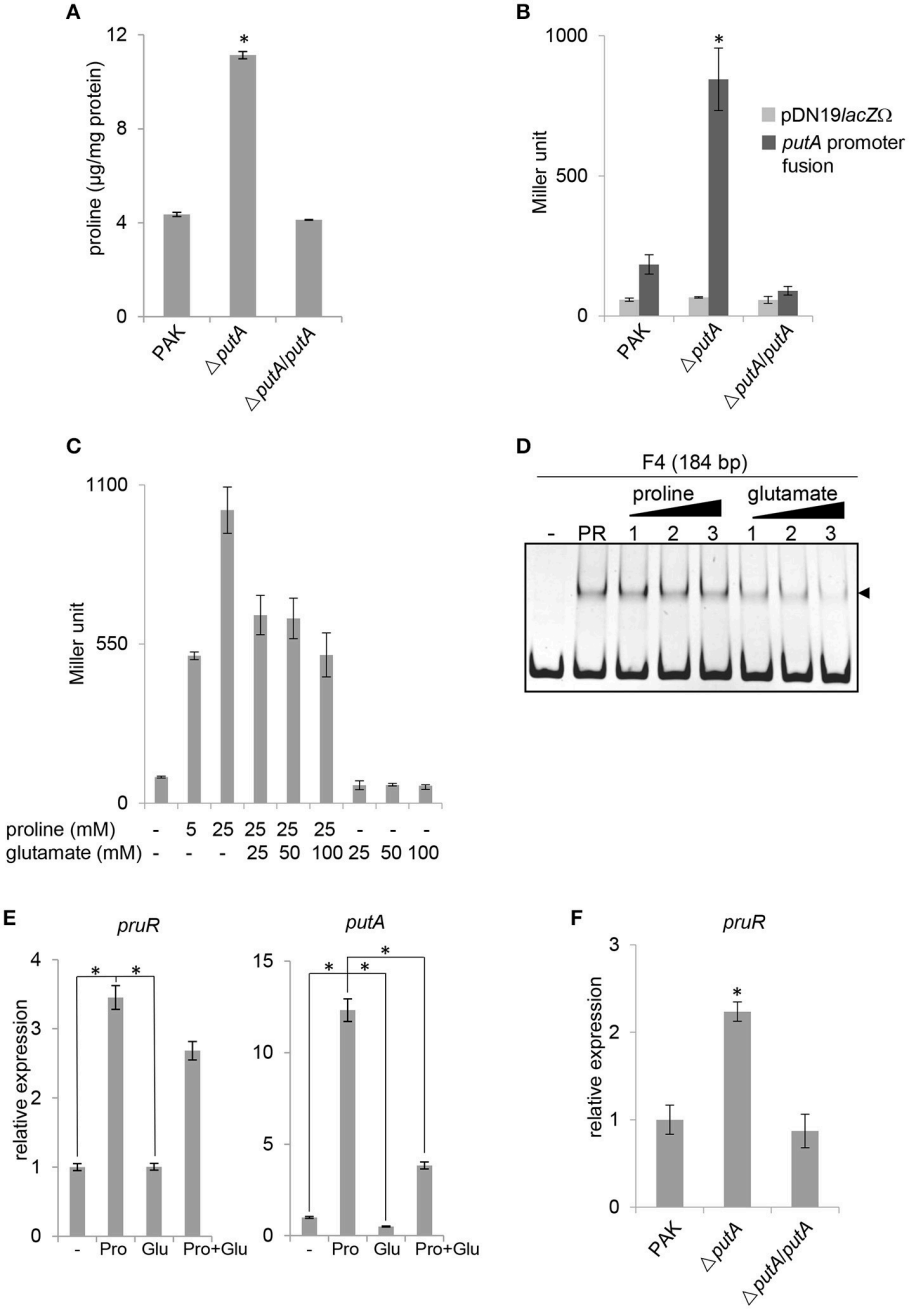
Proline and glutamate are the signals regulating the *putA* expression. **(A)** Proline concentration in bacterial cells. Indicated strains were cultured in LB broth for overnight. Proline concentration in bacterial cells was measured. The values reflect proline amount relative to total bacterial protein amount. Data indicate the means ± standard deviations from three independent experiments performed in triplicate. ^*^P < 0.01 compared to wild type PAK strain, or the Δ*putA*/*putA* complemented strain by student's *t*-test. **(B)** The *putA* expression is up-regulated in the Δ*putA* mutant strain. β-galactosidase assays were used to measure the transcriptional activities of *lacZ* reporter fusions. Data indicate the means ± standard deviations from three independent experiments performed in triplicate. ^*^P < 0.01 compared to wild type PAK strain, or the Δ*putA*/*putA* complemented strain, or the Δ*putA* mutant strain harboring pDN19*lacZ*Ω vector by student's *t*-test. **(C)** The effects of proline and glutamate on the *putA* expression. The wild type PAK strain was cultured in MMP medium supplemented with different concentrations of proline and glutamate at 37°C for overnight. β-galactosidase assays were used to measure the transcriptional activities of the *putA* promoter-*lacZ* fusions. Data indicate the means ± standard deviations from three independent experiments performed in triplicate. **(D)** EMSA for dose-dependent effects of proline and glutamate on the binding of PruR to the *putA* promoter. DNA fragment F4 (50 ng) was incubated alone (–) or with 2 μg rPruR (PR) or with 2 μg rPruR and proline or glutamate at different concentrations (lanes 1–3: 25, 50, and 100 mM, respectively). Shifted bands are indicated by an arrowhead. Black triangles show proportions of concentration of proline or glutamate. **(E)** The expression of *pruR* and *putA* in different media. RNA samples for quantitative RT-PCR were prepared from the wild type PAK strain cultured in different media at 37°C for overnight. –, MMP medium only; Pro, MMP medium supplemented with 25 mM proline; Glu, MMP medium supplemented with 25 mM glutamate; Pro+Glu, MMP medium supplemented with 25 mM proline and 25 mM glutamate. The expression of *pruR* or *putA* is normalized against that of 16S rRNA. Values relative to the amount cultured in MMP medium are shown. Data indicate the means ± standard deviations from three independent experiments performed in triplicate. ^*^P < 0.01 by student's *t*-test. **(F)** The expression of *pruR* in different strains. RNA samples for quantitative RT-PCR were prepared from indicated strains cultured in LB broth at 37°C for overnight. The *pruR* expression is normalized against that of 16S rRNA. Values relative to the amount of the wild type PAK strain are shown. Data indicate the means ± standard deviations from three independent experiments performed in triplicate. ^*^P < 0.01 compared to the wild type PAK strain or the Δ*putA*/*putA* complemented strain by student's *t*-test.

To determine the signals regulating the *putA* expression, we constructed reporter plasmid by inserting *putA* promoter upstream of a promoter-less *lacZ* gene in a pDN19*lacZ*Ω plasmid and transformed the resulting fusion construct into the wild type PAK strain, the Δ*putA* mutant strain or the Δ*putA*/*putA* complemented strain. These strains were cultured in LB broth at 37°C for overnight. Due to the lack of proline utilization, proline concentration was significantly higher in the Δ*putA* mutant strain than that in the wild type PAK strain or the Δ*putA*/*putA* complemented strain (Figure 6A). At the same time, the *putA* expression was activated as β-galactosidase activity was significantly increased in the Δ*putA* mutant strain (Figure 6B), suggesting the high proline concentration is an activation signal for *putA* expression.

To confirm the effect of proline on the *putA* expression, the wild type PAK strain was cultured in MMP medium supplemented with different concentration of proline. The addition of proline resulted in increased levels of β-galactosidase activity in a dose-dependent manner (Figure 6C and Figure S3). To further investigate the regulation of the *putA* expression, the effect of glutamate, which is the product of PutA enzyme activity, was examined. The high level of β-galactosidase activity induced by proline was repressed by the addition of glutamate (Figure 6C). However, adding proline into the binding reaction did not increase the affinity of rPruR to the *putA* promoter (Figure 6D). Meanwhile, glutamate released rPruR from the *putA* promoter in a dose-dependent manner (Figure 6D). The expression of *pruR* and *putA* was highly up-regulated in MMP medium supplemented with 25 mM proline, but did not show significant changes in MMP medium supplemented with 25 mM glutamate (Figure 6E). The expression of *pruR* showed no significant difference in MMP supplemented with 25 mM proline and in MMP supplemented with 25 mM proline and 25 mM glutamate. However, the expression of *putA* in MMP supplemented with both proline and glutamate was significantly lower than that in MMP supplemented with proline (Figure 6E). Furthermore, the expression of *pruR* was highly up-regulated in the Δ*putA* mutant strain compared to that of the wild type PAK strain or the Δ*putA*/*putA* complemented strain (Figure 6F). All together, these results indicated that high concentration of proline up-regulates *pruR* expression, which in turn activates *putA* expression, and high glutamate concentration reduces the affinity of PruR to *putA* promoter, then turns off the expression of *putA*. We also examined whether PruR is auto-regulated. Our EMSA and β-galactosidase assay results shown in Figure S2 demonstrated that PruR neither bound to its own promoter region, nor activated its expression.

### PruR affects *P. aeruginosa* virulence through the regulation of the *putA* expression

**Figure 7:**
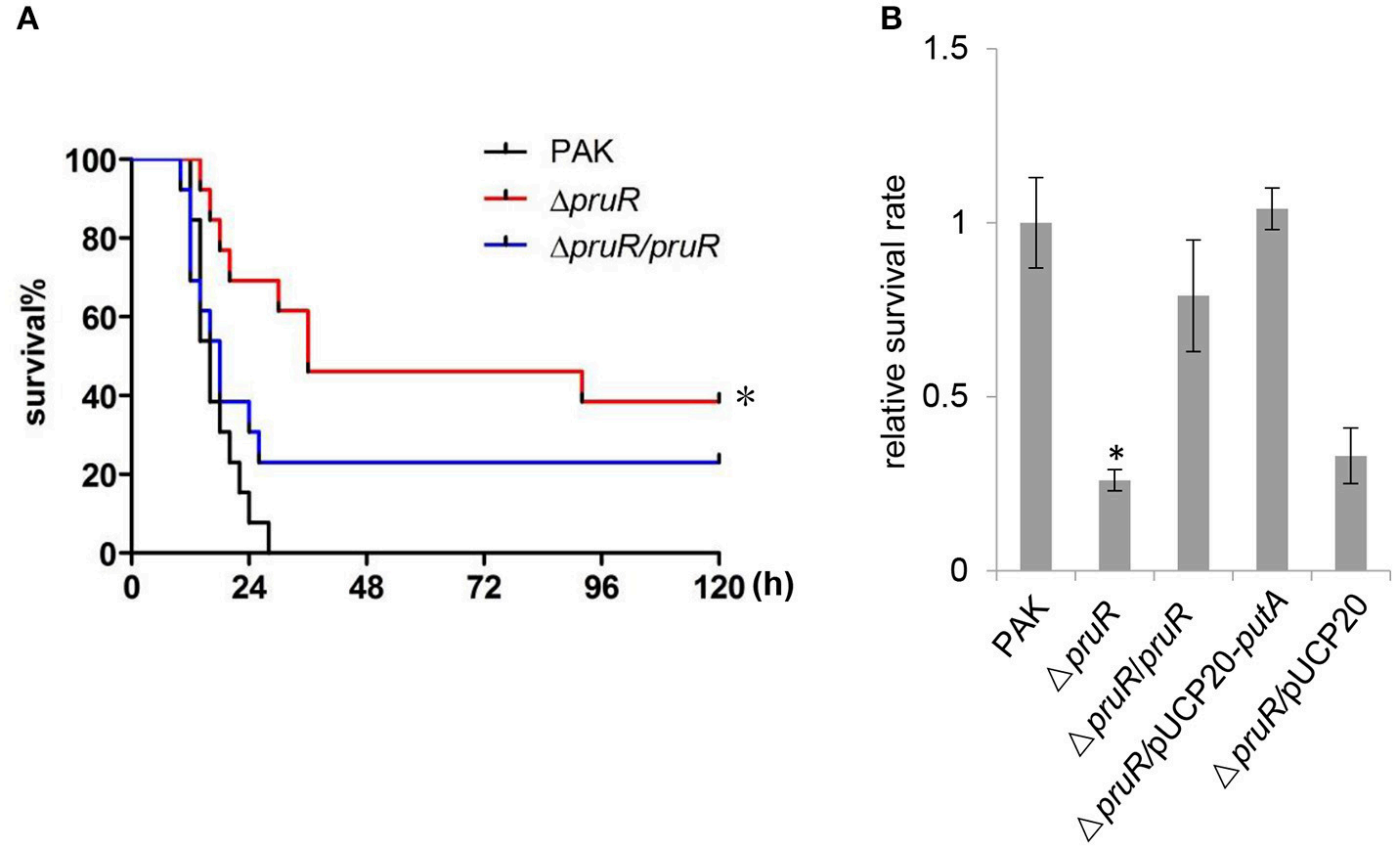
Role of PruR in *P. aeruginosa* infection in a mouse acute pneumonia model and bacterial resistance to oxidative stress. **(A)** Mice were inoculated intranasally with 4 × 10^7^ CFU bacteria of indicated strains. The mice were monitored for 5 days after the infection. The data were from 13 mice per strain. ^*^p < 0.01, compared to the wild type PAK strain or the Δ*pruR*/*pruR* complemented strain by log-rank (Mantel-Cox) test. **(B)** Indicated strains were treated with 0.3% H_2_O_2_ at 37°C for 15 min and the numbers of live bacteria were determined by serial dilution and plating. The values reflect bacterial numbers of each strain relative to that of the wild type PAK strain. Data indicate the means ± standard deviations from three independent experiments performed in triplicate. ^*^P < 0.01 compared to the wild type PAK strain, the Δ*pruR*/*pruR* complemented strain or the Δ*pruR*/pUCP20-*putA* PutA over-expression strain by student's *t*-test.

To evaluate the roles of PruR in the virulence of *P. aeruginosa*, we infected mice with the wild type PAK strain, a Δ*pruR* mutant strain or a Δ*pruR/pruR* complemented strain in the acute pneumonia model. Compared to the wild type PAK strain, the Δ*pruR* mutant strain caused significantly lower mortality (*P* < 0.01; Figure 7A). Complementation with a *pruR* gene driven by its native promoter restored the bacterial virulence (Figure 7A). The growth rates of these three strains showed no differences when cultured on LB broth or MMP medium supplemented with glucose and (NH_4_)_2_SO_4_ (Figure S4). These results indicated that PruR is required for the virulence of *P. aeruginosa* in the murine acute pneumonia model.

We further examined the resistance of the Δ*pruR* mutant strain to oxidative stress. The wild type PAK strain, the Δ*pruR* mutant strain or the Δ*pruR*/*pruR* complemented strain was treated with 0.3% H_2_O_2_ at 37°C for 15 min. Survival rate of the Δ*pruR* mutant strain was significantly lower than those of the wild type PAK strain and the Δ*pruR*/*pruR* complemented strain (Figure 7B). Overexpression of a *putA* gene rescued the Δ*pruR* mutant strain from the increased sensitivity to H_2_O_2_-mediated killing (Figure 7B). These experiments indicated that the *P. aeruginosa* PruR affects bacterial survival under oxidative stress through the regulation of the *putA* expression.

## Discussion

In this study, we demonstrated that PutA is required for the virulence of *P. aeruginosa* in a murine acute pneumonia model. Further experiments demonstrated that PruR directly binds to the *putA* promoter. High concentration of proline in bacteria up-regulates *pruR* expression. Then PruR activates *putA* expression. As a feedback regulation, glutamate produced by PutA turns off the *putA* expression. PruR affected bacterial virulence through the regulation of *putA* expression. It is for the first time to reveal that the proline utilization plays an important role in the pathogenesis of *P. aeruginosa*, as well as to describe the genetic regulation of the Put system in *P. aeruginosa*.

Proline is a multifunctional amino acid playing important roles in carbon and nitrogen metabolism, protein synthesis, and protection against various environmental stresses such as drought, osmotic stress, and oxidative stress (Barnett and Naylor, 1966; Csonka, 1981a; Wood, 1988; Chen and Dickman, 2005; Krishnan et al., 2008; Szabados and Savoure, 2010; Natarajan et al., 2012; Liang et al., 2013). Wild type *P. aeruginosa* exhibits higher resistance to H_2_O_2_ stress than the Δ*putA* mutant strain. And complementation of *putA* gene could restore oxidative stress protection. These results indicate that proline also provides resistance against oxidative stress in *P. aeruginosa* which is a PutA dependent phenomenon.

OxyR plays important roles in the regulation of oxidative stress responsive genes in *E. coli* and *P. aeruginosa* (Zheng et al., 2001; Jo et al., 2015). Oxidation of two conserved cysteine residues in OxyR results in formation of an intra-molecular disulfide bond, which leads to the binding of OxyR to promoters of target genes, then activates their expression (Jo et al., 2015). In *E. coli*, OxyR regulates the expression of responsive genes, such as *katG* (hydroperoxidase I), *ahpCF* (peroxiredoxin AhpCF), *grxA* (glutaredoxin I), and *trxC* (thioredoxin 2), to protect bacteria against ROS (Zheng et al., 2001). Under oxidative stress, proline utilization by PutA changes bacterial intracellular redox condition, which activates OxyR, leading to increased expression of *katG, grxA*, and *trxC* (Zhang et al., 2015). In *P. aeruginosa*, OxyR regulates the expression of *katA, katB, ahpB*, and *ahpCF* to defend against host produced ROS (Ochsner et al., 2000; Lee et al., 2005; Heo et al., 2010). Proline protection against oxidative stress of *P. aeruginosa* might also depend on OxyR regulation. This possibility is currently being investigated by our group.

In the absence of proline, PutA protein of *E. coli, S. typhimurium*, or *P. putida* represses the expression of *putA* gene and the divergent *putP* gene (Muro-Pastor and Maloy, 1995; Vílchez et al., 2000; Becker and Thomas, 2001). In contrast, *P. aeruginosa* PutA lacks the DNA binding domain and is unlikely auto-regulated. In *A. tumefaciens* and *R. capsulatus* PutR acts as a transcriptional activator of *putA*. Adding proline into culture medium leads to the transcriptional activation of *putA* through PutR. Subsequently PutR suppresses *putR* expression (Keuntje et al., 1995; Cho and Winans, 1996). However, *P. aeruginosa* does not encode PutR homolog. Our results showed that PruR binds to the *putA* promoter region and activates the *putA* expression. Proline did not change the affinity of PruR to the binding site, instead, high intracellular proline concentration up-regulated the *pruR* expression. Also we found that PruR is not auto-regulated. These results suggest that there might be other unknown mechanisms involved in proline signal detection and *pruR* regulation, which then controls *putA* expression.

In *B. abortus*, the two-component system NtrY/NtrX acts as a redox sensor sensing oxidative tension, and regulates the expression of nitrogen respiration enzymes (Carrica Mdel et al., 2012). In *E. chaffeensis*, NtrY/NtrX regulates the expression of PutA and GlnA, which converts glutamate to glutamine by nitrogen assimilation (Cheng et al., 2014). *P. aeruginosa* however does not encode NtrY/NtrX, but NtrB/NtrC, which is the homologous pair to NtrY/NtrX. In *E. coli*, the sensor kinase NtrB detects the concentration changes of α-ketoglutarate and glutamine through a signal transduction protein P(II) and phosphorylates the cognate response regulator NtrC, which then regulates the expression of nitrogen metabolism genes, including *glnA* (Ninfa et al., 2000; Dixon and Kahn, 2004; Lilja et al., 2004; Forchhammer, 2008; Hervás et al., 2009). In *P. aeruginosa* genome, P(II) protein is encoded by *glnK* gene. Proline is an important nitrogen source for bacterial growth (Wood, 1981; Kohl et al., 1988; Nagata et al., 2003). Proline accumulation might affect the concentration of α-ketoglutarate and glutamine, which can be converted from proline. P(II) might detect these changes and activate *pruR* expression through the NtrB/NtrC system. Our group is investigating this hypothesis now.

The AraC/XylS family proteins consist of two domains, a non-conserved domain, which seems to be involved in effector/signal reorganization and dimerization, and a conserved DNA binding domain that contains two helix–turn–helix DNA binding motifs (Gallegos et al., 1997). *E. coli* AraC binds to *araFGH* promoter, facilitates recruitment of RNA polymerase and isomerization to open complex, and prevents improper binding of RNA polymerase (Johnson and Schleif, 2000). Our results showed that *P. aeruginosa* PruR binding site was located from −71 to −41 counting from transcriptional start site in the *putA* promoter region, which is right upstream of the −35 box. Interestingly PruR binding was affected by glutamate concentration. It is possible that PruR initiates *putA* expression by RNA polymerase recruitment, and the non-conserved domain of PruR senses the intracellular glutamate concentration. Our results showed that high concentration of glutamate did not release PruR from the *putA* promoter completely, suggesting there might be additional regulatory mechanisms involved. To fully elucidate the mechanism of PruR regulation on PutA expression, further studies on the interaction among PruR, RNA polymerase, and glutamate are warranted.

## Author contributions

ZC, RZ, and XF: conceived and designed the experiments and wrote the paper; RZ, XF, XW, XP, CL, RS, YJ, and FB: performed the experiments; ZC, RZ, XF, SJ, and WW: analyzed the data.

### Conflict of interest statement

The authors declare that the research was conducted in the absence of any commercial or financial relationships that could be construed as a potential conflict of interest.

## Supplementary material

Table S1 Bacterial strains used in this study.

Table S2 Plasmids used in this study.

Table S3 Oligonucleotides used in this study.

Figure S1 The growth rate of the Δ*putA* mutant strain shows no differences to that of the wild type PAK strain or the Δ*putA/putA* complemented strain. Indicated strains were cultured in **(A)** LB broth or **(B)** MMP medium containing 1.0% (w/v) glucose and 0.1% (w/v) (NH_4_)_2_SO_4_ at 37°C.

Figure S2 PruR does not activate the expression of *PA0781* and *pruR*. **(A)** PruR does not activate the expression of *PA0781* and *pruR*. β-galactosidase assays were used to measure the transcriptional activities of *lacZ* reporter fusions. Data indicate the means ± standard deviations from three independent experiments performed in triplicate. Western blot analyses of samples from the β-galactosidase assays were performed using an anti-His-tag antibody to verify the expression of rPruR. The blots below the graphs are representative blots for three independent experiments. The position of rPruR are indicated by arrowheads. **(B)** PruR does not bind to the *pruR* promoter. Lane 1, DNA probe (50 ng); lane 2, DNA probe incubated with rPruR (2 μg). The length (bp) of the fragment is shown above the panel. The gel was stained with ethidium bromide.

Figure S3 The effects of proline on the *putA* expression. The wild type PAK strain was cultured in MMP medium supplemented with different concentrations of proline at 37°C for overnight. β-galactosidase assays were used to measure the transcriptional activities of the *putA* promoter-*lacZ* fusions. Data indicate the means ± standard deviations from three independent experiments performed in triplicate.

Figure S4 The growth rate of the Δ*pruR* mutant strain shows no difference to that of the wild type PAK strain or the Δ*pruR/pruR* complemented strain. Indicated strains were cultured in **(A)** LB broth or **(B)** MMP medium containing 1.0% (w/v) glucose and 0.1% (w/v) (NH_4_)_2_SO_4_ at 37°C.
