# Supplementary material for: PutA Is Required for Virulence and Regulated by PruR in Pseudomonas aeruginosa
Source: Front Microbiol. 2018 Mar 26;9:548. doi: 10.3389/fmicb.2018.00548 (PMC5879082; doi:10.3389/fmicb.2018.00548)
Supplement: Table S1 — Bacterial strains used in this study. [file Table1.DOCX]

**Table S1. Bacterial strains used in this study.**

| Strain | Characteristics or function | Source/ Reference |
| --- | --- | --- |
| ***E. coli*** |  |  |
| DH5α | F^-^, φ80d*lacZ*ΔM15, Δ(*lacZYA-argF*)U169, *deoR*, *recA1*, *endA1*, *hsdR17*(*r*_k_^-^,*m*_k_^+^), *phoA*, *supE44*, λ^-^, *thi-1*, *gyrA96*, *relA1* | TransGen |
| S17-1 | *recA, pro, hsdR*, RP4-2-Tc::Mu-Km::Tn7 | Simon et al., 1983 |
| SI7-1/pEX18Tc-△*putA*::Gm | S17-1 containing pEX18Tc-△*putA*::Gm; Gm^r^ | This study |
| SI7-1/pEX18Tc-△*pruR*::Gm | S17-1 containing pEX18Tc-△*pruR*::Gm; Gm^r^ | This study |
| BL21(DE3)/pPruR | BL21(DE3) expressing PruR; Kan^r^ | This study |
| BL21(DE3)/pET-28a(+) | BL21(DE3) containing pET-28a(+) vector; Kan^r^ | This study |
| BL21(DE3)/pPruR/pACYC184*putA-lacZ* | BL21(DE3)/pPruR containing pACYC184*putA-lacZ*; Kan^r^ , Cam^r^ | This study |
| BL21(DE3)/pET-28a(+)/  pACYC184*putA-lacZ* | BL21(DE3)/pET-28a(+) containing pACYC184*putA-lacZ*; Kan^r^ , Cam^r^ | This study |
| ***P. aeruginosa*** |  |  |
| PAK | Wild type strain of *Pseudomonas aeruginosa* | Weng et al., 2016 |
| PAK △*putA* | *putA* deletion mutant of PAK | This study |
| PAK △*pruR* | *pruR* deletion mutant of PAK | This study |
| PAK △*putA/putA* | PAK △*putA* complemented by chromosome inserted *putA* gene | This study |
| PAK △*pruR/pruR* | PAK △*pruR* complemented by chromosome inserted *pruR* gene | This study |
| PAK △*pruR/*pUCP20 | PAK △*pruR* containing pUCP20 | This study |
| PAK △*pruR/*pUCP20-*putA* | PAK △*pruR* overexpressing PutA | This study |
| PAK/pDN19*lacZ*Ω | PAK containing pDN19*lacZ*Ω | This study |
| PAK/pDN19*putA-lacZ*Ω | PAK containing pDN19*putA-lacZ*Ω | This study |
| PAK △*putA*/pDN19*lacZ*Ω | PAK △*putA* containing pDN19*lacZ*Ω | This study |
| PAK △*putA*/pDN19*putA-lacZ*Ω | PAK △*putA* containing pDN19*putA-lacZ*Ω | This study |
| PAK △*putA/putA/*pDN19*lacZ*Ω | PAK △*putA/putA* containing pDN19*lacZ*Ω | This study |
| PAK △*putA/putA/*pDN19*putA-lacZ*Ω | PAK △*putA/putA* containing pDN19*putA-lacZ*Ω | This study |

Simon, R., Priefer, U., and Pühler, A. (1983). A broad host range mobilization system for *in vivo* genetic engineering: transposon mutagenesis in gram negative bacteria. *Nat Biotechnol* 1, 784-791.

Weng, Y., Chen, F., Liu, Y., Zhao, Q., Chen, R., Pan, X., et al. (2016). *Pseudomonas aeruginosa* enolase influences bacterial tolerance to oxidative stresses and virulence. *Front Microbiol* 7, 1999.
