# Supplementary material for: PutA Is Required for Virulence and Regulated by PruR in Pseudomonas aeruginosa
Source: Front Microbiol. 2018 Mar 26;9:548. doi: 10.3389/fmicb.2018.00548 (PMC5879082; doi:10.3389/fmicb.2018.00548)
Supplement: Table S2 — Plasmids used in this study. [file Table2.DOCX]

**Table S2. Plasmids used in this study.**

| Plasmid | Characteristics or function | Source/ Reference |
| --- | --- | --- |
| pEX18Tc | Broad-host-range gene replacement vector; Tc^r^, *oriT*^+^, *sacB*^+^ | Hoang et al., 1998 |
| pEX18Tc-△*putA*::Gm | *putA* gene of PAK deletion on pEX18Tc; Gm^r^, Tc^r^ | This study |
| pEX18Tc-△*pruR*::Gm | *pruR* gene of PAK deletion on pEX18Tc; Gm^r^, Tc^r^ | This study |
| pUC18T-mini­-Tn7T-Gm | For gene insertion in chromosome; Gm^r^ | Choi and Schweizer, 2006 |
| pUC18T-mini­-Tn7T-Gm-*putA* | pUC18T-mini­-Tn7T-Gm with *putA* gene driven by its native promoter for chromosomal insertion; Gm^r^ | This study |
| pUC18T-mini-Tn7T-Gm-*pruR* | pUC18T-mini­-Tn7T-Gm with *pruR* gene driven by its native promoter for chromosomal insertion; Gm^r^ | This study |
| pTNS3 | Helper plasmid; Amp^r^ | Choi and Schweizer, 2006 |
| pET-28a(+) | Clone vector; Kan^r^ | Novagen |
| pPruR | pET-28a(+) harboring *pruR* gene; Kan^r^ | This study |
| pACYC184*lacZ* | pACYC184 harboring promoterless *lacZ* gene; Cam^r^ | This study |
| pACYC184*putA-lacZ* | pACYC184*lacZ* harboring *putA* promoter -*lacZ* fusion; Cam^r^ | This study |
| pDN19*lacZ*Ω | Promoterless *lacZ* fusion vector; Sp^r^, Sm^r^, Tc^r^ | Weng et al., 2016 |
| pDN19*putA-lacZ*Ω | pDN19*lacZ*Ω harboring *putA* promoter-*lacZ* fusion; Sp^r^, Sm^r^, Tc^r^ | This study |
| pUCP20 | Clone vector; Cb^r^ | Choi and Schweizer, 2006 |
| pUCP20-*putA* | pUCP20 harboring *putA* gene; Cb^r^ | This study |

Hoang, T.T., Karkhoff-Schweizer, R.R., Kutchma, A.J., and Schweizer, H.P. (1998). A broad-host-range Flp-FRT recombination system for site-specific excision of chromosomally-located DNA sequences: application for isolation of unmarked *Pseudomonas aeruginosa* mutants. *Gene* 212(1), 77-86.

Choi, K.H., and Schweizer, H.P. (2006). Mini-Tn7 insertion in bacteria with single attTn7 sites: example *Pseudomonas aeruginosa*. *Nat Protoc* 1(1), 153-161.

Weng, Y., Chen, F., Liu, Y., Zhao, Q., Chen, R., Pan, X., et al. (2016). *Pseudomonas aeruginosa* enolase influences bacterial tolerance to oxidative stresses and virulence. *Front Microbiol* 7, 1999.
