# Supplementary material for: PutA Is Required for Virulence and Regulated by PruR in Pseudomonas aeruginosa
Source: Front Microbiol. 2018 Mar 26;9:548. doi: 10.3389/fmicb.2018.00548 (PMC5879082; doi:10.3389/fmicb.2018.00548)
Supplement: Table S3 — Oligonucleotides used in this study. [file Table3.DOCX]

**Table S3. Oligonucleotides used in this study.**

| Gene/Fragment | Sequence 5´→3´ |
| --- | --- |
| **Gene knockout and complementation** | |
| *putA* | UF^a^: TACTCAGAATTCGACGGGTTCGCTGGGGAT^b^  UR: TACTCAGGATCCTTTGAACATCACGCCCTCCT |
| *putA* | DF: TACTCAGGATCCCGTCGCTGCTGTCGCTGG  DR: TACTCAAAGCTTCGGGTTGAACAGGATCTTGG |
| *putA* | F: TACTCAAAGCTTCCTGGGAGGGCAATTCGCTGGG  R: TACTCAGGATCCTCGTCCTTTATGGTCCGTTC |
| *pruR* | UF: CCCGAATTCGCCGATCTTGCGGTTGACCT  UR: CCCGGATCCTGGTCGGGCAGACGGATGAG |
| *pruR* | DF: CCCGGATCCGCTGACGCCGAAGTCGCTGC  DR: CCCAAGCTTCGCACACGGAAACCGACAGC |
| *pruR* | F: CCCAAGCTTTGCTCGGGCAGGCTGTTGAC  R: CCCGGATCCTTATCCGTCCTACACCACTC |
| **Protein expression** |  |
| PutA | F: CCCGGATCCATGTTCAAAGCCAGTCACGT |
|  | R: CCC**AAGCTT**TCACTCGGCGTCGGCCAGCG |
| PruR | F: CCCGGATCCATGTTCGACACCCGCCTCA |
|  | R: CCCAAGCTTTCAGCCCTTGCGCAGCGA |
| ***lacZ* fusion** |  |
| *putA*F1 | F: CCCTCTAGATTCCCCCGAAACAATCAGG  R: CCCGGATCCCACGCCCTCCTCTTGTGGA |
| *putA*F2 | F: CCCTCTAGAATCGGCGTATTTATTGGGATG  R: CCCGGATCCCACGCCCTCCTCTTGTGGA |
| *putA*F3 | F: CCCTCTAGACCCTCTCGCAGCCACTAC  R: CCCGGATCCCACGCCCTCCTCTTGTGGA |
| *putA*F7 | F: CCCTCTAGACCAGGAGTTTTCCGCAAAAACTC  R: CCCGGATCCCACGCCCTCCTCTTGTGGA |
| *putA*F7MU | F: CCCTCTAGAATAGGAGTTTTCCGCAAAAACTC  R: CCCGGATCCCACGCCCTCCTCTTGTGGA |
| *putA*F8 | F: CCCTCTAGATTCGCAAGAATCGCCCCTCTCGCA  R: CCCGGATCCCACGCCCTCCTCTTGTGGA |
| *pruR* | F: CCCTCTAGACGGAGGATTGTCCATGAA |
|  | R: CCCGGATCCGAACGGCAACTGCTCAAT |
| *PA0781* | F: CCCGGATCCTGCGGGCTCCATGGTCATGG |
|  | R: CCCTCTAGACACGCCCTCCTCTTGTGGA |
| *putA* | **F: TACTCAGAATTC**TTCCCCCGAAACAATCAGG |
|  | R: **TACTCA**GGATCCCACGCCCTCCTCTTGTGGA |
| ***putA* promoter region for EMSA** | |
| F1 | F: TTCCCCCGAAACAATCAGG |
|  | R: CACGCCCTCCTCTTGTGGA |
| F2 | F: ATCGGCGTATTTATTGGGATG |
|  | R: CACGCCCTCCTCTTGTGGA |
| F3 | F: CCCTCTCGCAGCCACTAC |
|  | R: CACGCCCTCCTCTTGTGGA |
| F4 | F: ATCGGCGTATTTATTGGGATG^c^ |
|  | R: GCGATTCTTGCGAAAGACTC |
| F5 | F: ATCGGCGTATTTATTGGGATG |
|  | R: CTTTCCCGTTCCTCTTCGC |
| F6^d^ | F: TTCCGCAGCCAGGAGTTTTCCGCAAAAACTCCAGAGTC TTTCGCAAGAATCGC |
|  | R: GCGATTCTTGCGAAAGACTCTGGAGTTTTTGCGGAAAA CTCCTGGCTGCGGAA |
| *pruR* | F: CGGAGGATTGTCCATGAA |
|  | R: GAACGGCAACTGCTCAAT |
| **Gene-specific quantitative RT-PCR** | |
| *putA* | F: ATGAACCAGGCGATGAAG |
|  | R: ATGTCGAAGGAATAGGTGTAG |
| *pruR* | F: CATATCGTGCGGAACCTG |
|  | R: GACGCTGTCCTTGAACTG |
| 16S rRNA | F: AAGGTCTTCGGATTGTAA  R: GTGCTTATTCTGTTGGTAA |

^a^F, forward; R, reverse; U, upstream of specific gene; D, downstream of specific gene. ^b^Enzyme sites are underlined.

^c^This primer was also labeled with FAM and used to amplify the fragment for DNase I footprint assay.

^d^This set of oligonucleotides was annealed for EMSA.
