# Supplementary figures and images for: PutA Is Required for Virulence and Regulated by PruR in Pseudomonas aeruginosa
Source: Front Microbiol. 2018 Mar 26;9:548. doi: 10.3389/fmicb.2018.00548 (PMC5879082; doi:10.3389/fmicb.2018.00548)

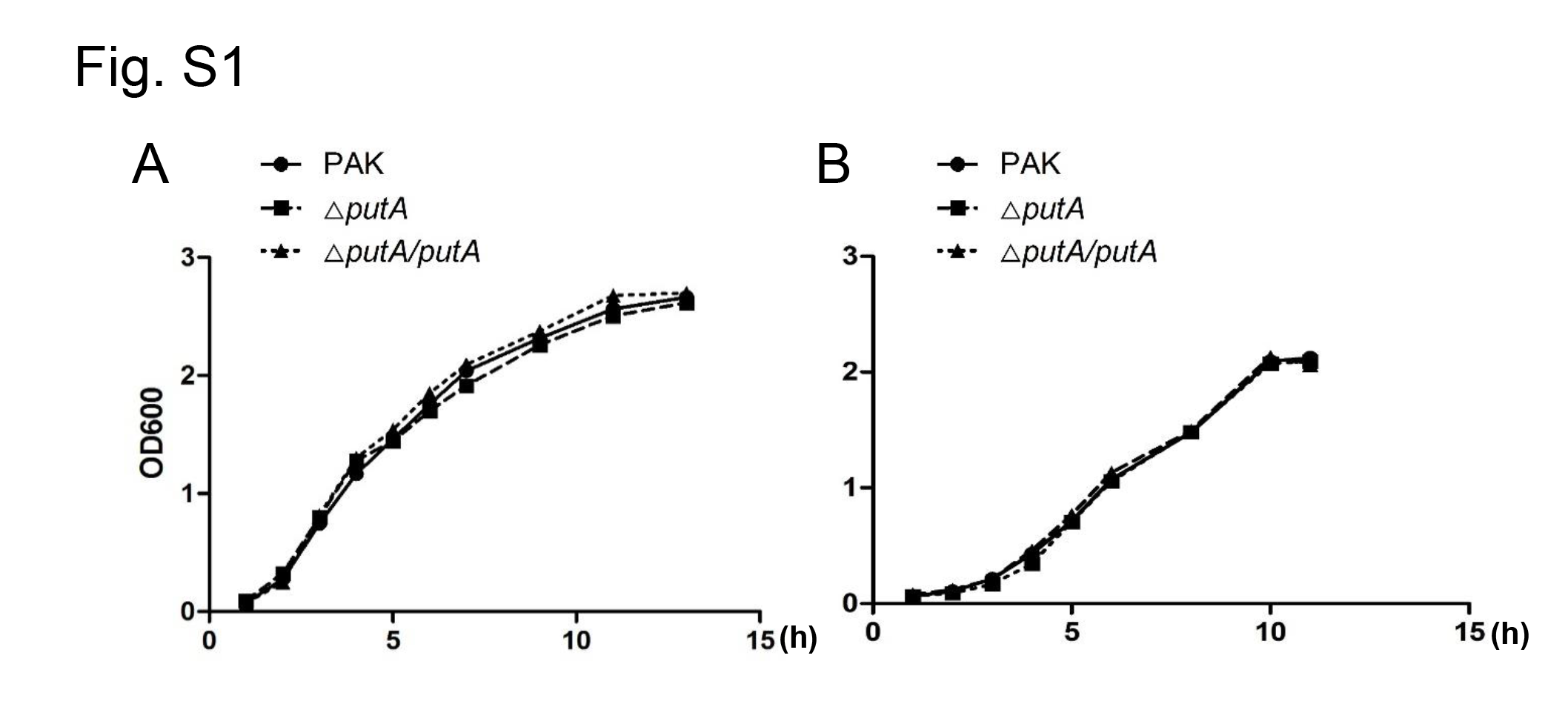

Supplement: Figure S1 — The growth rate of the ΔputA mutant strain shows no differences to that of the wild type PAK strain or the ΔputA/putA complemented strain. Indicated strains were cultured in (A) LB broth or (B) MMP medium containing 1.0% (w/v) glucose and 0.1% (w/v) (NH4)2SO4 at 37°C. [file Image1.TIF]

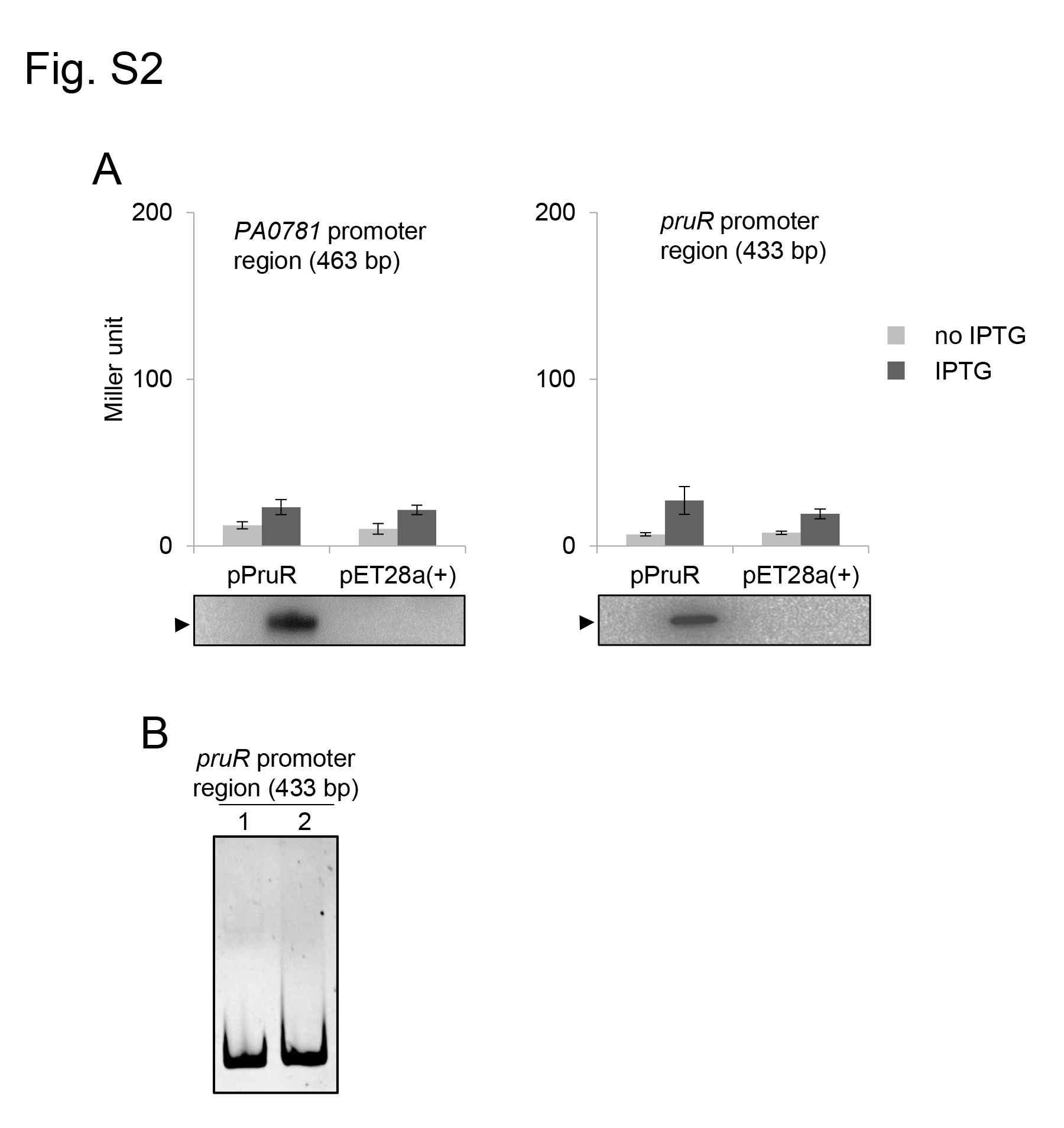

Supplement: Figure S2 — PruR does not activate the expression of PA0781 and pruR. (A) PruR does not activate the expression of PA0781 and pruR. β-galactosidase assays were used to measure the transcriptional activities of lacZ reporter fusions. Data indicate the means ± standard deviations from three independent experiments performed in triplicate. Western blot analyses of samples from the β-galactosidase assays were performed using an anti-His-tag antibody to verify the expression of rPruR. The blots below the graphs are representative blots for three independent experiments. The position of rPruR are indicated by arrowheads. (B) PruR does not bind to the pruR promoter. Lane 1, DNA probe (50 ng); lane 2, DNA probe incubated with rPruR (2 μg). The length (bp) of the fragment is shown above the panel. The gel was stained with ethidium bromide. [file Image2.TIF]

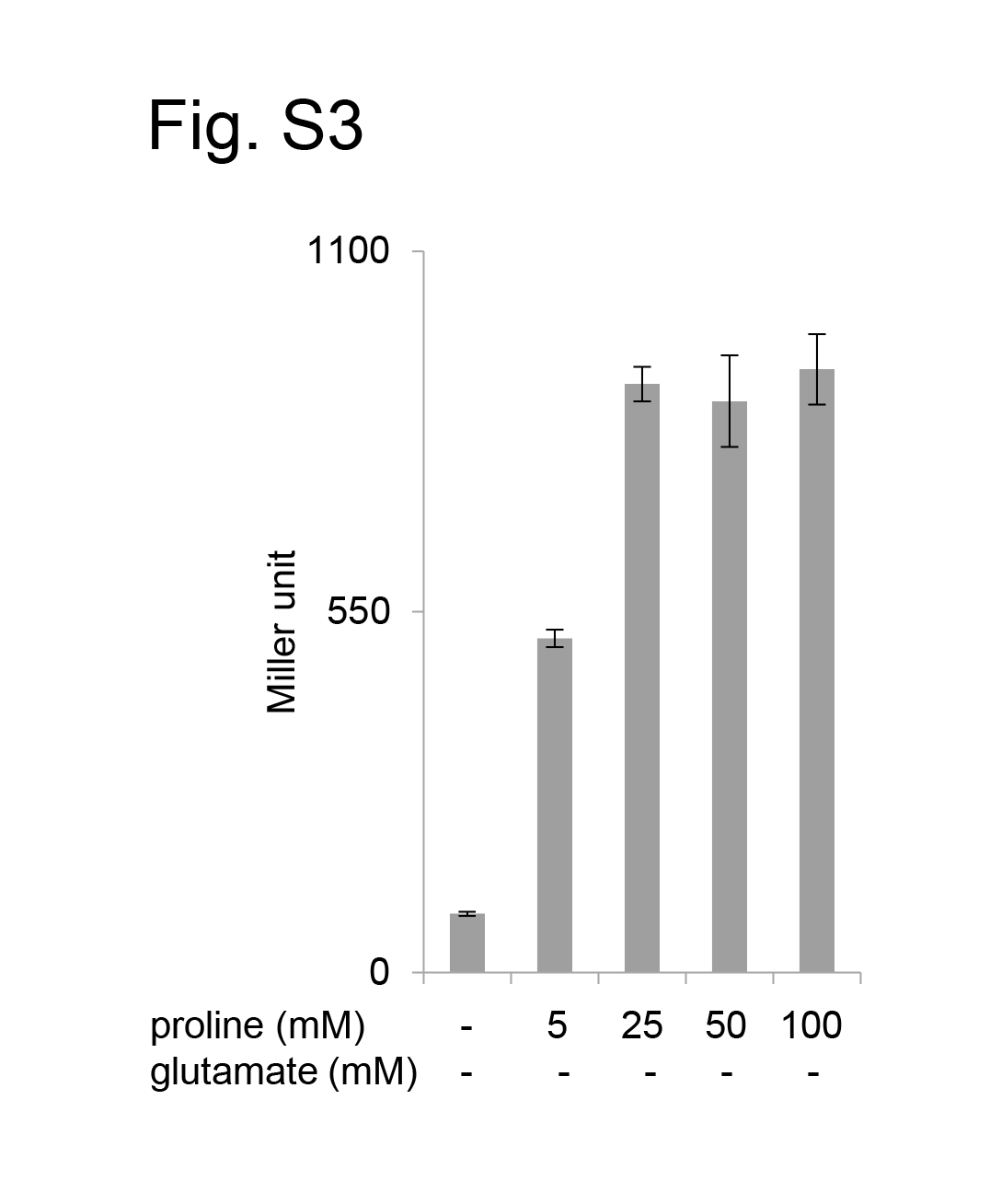

Supplement: Figure S3 — The effects of proline on the putA expression. The wild type PAK strain was cultured in MMP medium supplemented with different concentrations of proline at 37°C for overnight. β-galactosidase assays were used to measure the transcriptional activities of the putA promoter-lacZ fusions. Data indicate the means ± standard deviations from three independent experiments performed in triplicate. [file Image3.TIF]

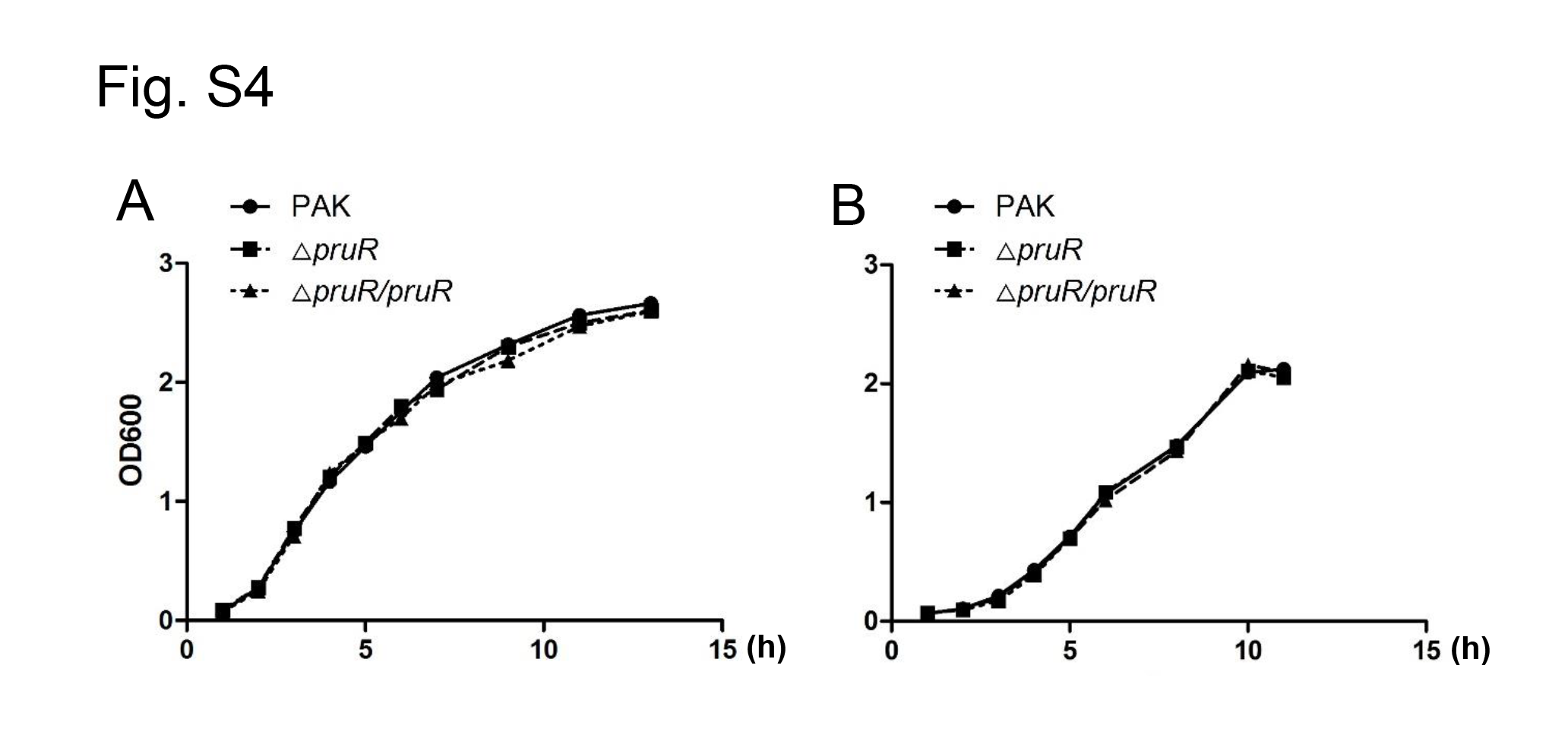

Supplement: Figure S4 — The growth rate of the ΔpruR mutant strain shows no difference to that of the wild type PAK strain or the ΔpruR/pruR complemented strain. Indicated strains were cultured in (A) LB broth or (B) MMP medium containing 1.0% (w/v) glucose and 0.1% (w/v) (NH4)2SO4 at 37°C. [file Image4.TIF]
